# Rise in Babesiosis Cases, Pennsylvania, USA, 2005–2018

**DOI:** 10.3201/eid2608.191293

**Published:** 2020-08

**Authors:** David Ingram, Tonya Crook

**Affiliations:** Penn State Health Milton S. Hershey Medical Center, Hershey, Pennsylvania, USA

**Keywords:** babesiosis, *Babesia*, tickborne diseases, parasitic infection, Pennsylvania, United States, parasites, vector-borne infections

## Abstract

Babesiosis is an emerging infection in the state of Pennsylvania, and clinicians need to be made aware of its clinical manifestations as well as the risk factors associated with severe disease. Before 2010, our tertiary academic center in central Pennsylvania previously saw zero cases of babesiosis. We saw our first confirmed case of *Babesia* infection acquired in Pennsylvania in 2011; we recorded 2 confirmed cases in 2017 and 4 confirmed cases in 2018. All 4 cases from 2018 were thought to be acquired in southcentral Pennsylvania counties, whereas prior reports of cases were predominately in the southeast and northeast counties of the state.

Babesiosis, a tickborne infection caused by protozoan parasites that infect erythrocytes, has been identified as an emerging infection of concern in the state of Pennsylvania, USA (*1*–*4*). Infection with *Babesia* parasites can cause a range of symptoms, including fever, myalgias, and fatigue. Although many patients are asymptomatic, the infection can be severe in some persons, especially those who are >50 years of age, immunosuppressed, or asplenic (*1*,*5*,*6*).

The most common species known to cause human infection in the United States is *Babesia microti*, which is transmitted by the *Ixodes scapularis* tick (blacklegged tick). Transmission can also occur through blood transfusion; *B. microti* is currently the most common pathogen transmitted through the blood supply in the United States (*7*,*8*). The highest incidence of human infection has been reported in the Northeast and upper Midwest states (*9*). Until fairly recently, few cases of *B. microti* infection have occurred in Pennsylvania. However, new data suggest not only increased prevalence of ticks harboring *B. microti* within the state (*10*) but also a rise in the number of cases seen in clinical practice (*2*–*4*).

Babesiosis is not a mandatory reportable infection in Pennsylvania; however, the Pennsylvania Department of Health does receive reports of cases of babesiosis from healthcare providers that elect to do so. Among these recounted cases, the department has seen a 20-fold increase in the past 12 years (E. Negrón, Pennsylvania Department of Health, pers. comm., 2018 Aug 1). A similar trend was documented in a recent study from February 2019 involving a 4-hospital system in southeastern Pennsylvania, where clinicians saw an increase in cases from <7 cases annually during 2008–2014 to 26 cases in 2015 (*4*).

We have suspected a similar increase in the number of cases at our institution, Penn State Milton S. Hershey Medical Center (Hershey, PA, USA), which is a tertiary academic center located in central Pennsylvania. We performed a retrospective review of all of the confirmed cases of babesiosis at our institution for the period 2005–2018 to determine whether we were truly seeing an increased number of cases and to highlight the demographic and clinical characteristics of these patients.

## Methods

We obtained a list of all patients who had International Classification of Diseases, 9th Revision (ICD-9) (088.82), and International Classification of Diseases, 10th Revision (ICD-10) (B60.0), diagnostic codes for babesiosis as well as patients that had *Babesia* serologic tests ordered at Hershey Medical Center during 2005–2018. The list consisted of 352 patient encounters, some of which were duplicates. We retrospectively chart reviewed each patient encounter to identify confirmed cases of babesiosis. Only patients who met Centers for Disease Control and Prevention criteria for confirmed cases of babesiosis were included in our study (Table 1): patients who had confirmatory laboratory results (i.e., parasite seen on peripheral smear, positive PCR from blood, or both) and met >1 of the objective or subjective clinical evidence criteria. Although we did not use positive serologic test results as a part of our diagnostic criteria, identifying patients who had serologic tests ordered was used to increase the number of patients in our initial cohort. We noted inconsistencies in the way blood smears and PCR testing were ordered in our electronic medical records, and we wanted to ensure we did not miss any cases. 

**Table 1:** Centers for Disease Control and Prevention criteria for diagnosis of confirmed cases of babesiosis, 2011*

Laboratory criteria for diagnosis	Clinical criteria for diagnosis
Identification of intraerythrocytic *Babesia* organisms by light microscopy in a Giemsa, Wright, or Wright-Giemsa–stained blood smear; OR detection of *Babesia microti* DNA in a whole blood specimen by PCR; OR detection of *Babesia* spp. genomic sequences in a whole blood specimen by nucleic acid amplification; OR Isolation of *Babesia* organisms from a whole blood specimen by animal inoculation.	Objective: >1 of the following: fever, anemia, or thrombocytopenia
	Subjective: >1 of the following: chills, sweats, headache, myalgia, or arthralgia.

*Includes cases that have confirmatory laboratory results and meet >1 of the objective or subjective clinical evidence criteria, regardless of the mode of transmission (can include clinically manifest cases in transfusion recipients or blood donors). Full case definition available at https://wwwn.cdc.gov/nndss/conditions/babesiosis/case-definition/2011.

The *Babesia* serologic testing used at Hershey Medical Center is the indirect fluorescent antibody (IFA) test. This test is specific for *B. microti* species. We considered a titer >1:256 to be positive on the basis of the titer value determined to be supportive of the diagnosis of babesiosis according to Centers for Disease Control and Prevention laboratory criteria (*11*). From our initial list of 352 patient encounters, we identified 8 cases of confirmed babesiosis. We maintained demographic, clinical, and laboratory data in a REDCap Electronic Database (*12*). Research protocols were reviewed and approved by the Penn State College of Medicine Institutional Review Board.

## The Patients

### Demographic Characteristics

**Figure 1:**
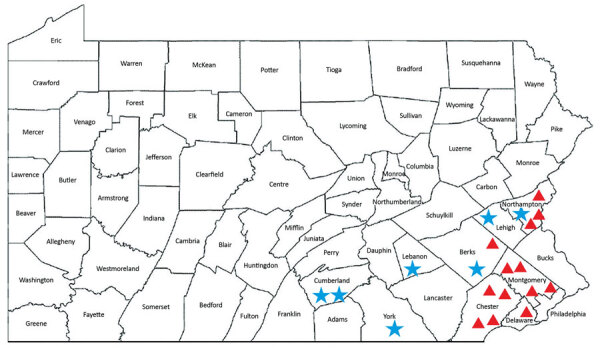
Counties where confirmed babesiosis cases were thought to have been acquired during 2011–2018 according to previous studies (*2**–**4*) compared with cases seen at Penn State Health Milton S. Hershey Medical Center, Hershey, Pennsylvania, USA, during 2005–2018. Red triangles indicate cases from previous studies (not all cases shown); blue stars indicate cases seen at Hershey Medical Center.

Of the 8 confirmed cases of babesiosis seen at our institution, 7 of the cases were acquired in the state of Pennsylvania (Table 2). One case was thought to have been acquired in Massachusetts. Of the 7 cases in Pennsylvania, more than half (4/7) were acquired in south-central Pennsylvania counties; 2 cases were from northeast counties, and 1 case was from a southeast county (Figure 1). No cases were reported during 2005–2010, 1 case was reported in 2011, 1 case in 2015, 2 cases in 2017, and 4 cases in 2018. All cases were diagnosed during the summer months. All but 1 of the patients were >60 years of age at the time of diagnosis. The median age at time of diagnosis was 70 years (range 20–77 years), and 75% of the patients were male. Only 1 patient reported a history of tick bite preceding infection. Six of 8 patients reported history of outdoor activity before seeking care. None of the patients had history of recent blood transfusion. 

**Table 2:** Demographic characteristics of 8 patients with confirmed babesiosis, Penn State Health Milton S. Hershey Medical Center, Hershey, Pennsylvania, USA, 2005–2018

Patient no.	Age, y/sex	Date patient sought care	Location of infection acquisition*	History of tick bite	History of outdoor activity	History of recent transfusion
1	77/F	2011 Jul 1	Northampton County	No	No	No
2	75/M	2015 Aug 4	Berks County	No	Yes	No
3	70/M	2017 Jun 30	Massachusetts	Yes	Yes	No
4	75/M	2017 Jul 19	Lehigh County	No	Yes	No
5	63/M	2018 Jun 19	York County	No	Yes	No
6	20/M	2018 Jun 27	Cumberland County	No	Yes	No
7	70/F	2018 Jul 2	Lebanon County	No	No	Unknown
8	65/M	2018 Jul 21	Cumberland County	No	Yes	No

*All counties are in Pennsylvania.

### Clinical Manifestations

The cohort included 2 patients with a history of splenectomy and 1 patient with a history of diabetes. Most of the patients were immunocompetent. None of the patients were on immunosuppressive therapy. No HIV patients or posttransplant patients were in the cohort. Most patients reported fever (6/8) and malaise (5/8). Other symptoms included myalgias or arthralgias (2/8), anorexia (2/8), rash (1/8), headache (1/8), nausea or vomiting (1/8), diarrhea (1/8), and respiratory failure (1/8). The average time from symptom onset to diagnosis was 9.7 days. The most common laboratory abnormalities seen were anemia, thrombocytopenia, transaminitis, and hyperbilirubinemia. Anemia was seen in all of the patients; average hemoglobin level was 9.8 g/dL (reference range 13–16 g/dL) (Table 3). Thrombocytopenia was seen in 7/8 patients; average platelet count was 90.8 × 10^9^/L (reference range 150–350 × 10^9^/L). Most of the patients (6/8) had platelet counts of <75. Elevated alanine aminotransferase and aspartate aminotransferase were seen in 7/8 patients; average alanine aminotransferase was 72 U/L (reference range 0–40 U/L) and average aspartate aminotransferase 176.6 U/L (reference range 0–40 U/L). Hyperbilirubinemia was also seen in 7/8 patients; average bilirubin level was 6.4 mg/dL (reference range 0–1.2 mg/dL). 

**Table 3:** Laboratory results for 8 patients with confirmed babesiosis, Penn State Health Milton S. Hershey Medical Center, Hershey, Pennsylvania, USA, 2005–2018

Test	Average (range)	Reference range
Leukocyte, × 10^9^ cells/L	8.7 (3.5–17.5)	4–10.4
Hemoglobin, g/dL	9.8 (6.3–11.9)	13–17
Platelets, × 10^9^ /L	90.8 (26–307)	150–350
Alanine aminotransferase, U/L	72 (23–185)	0–41
Aspartate aminotransferase, U/L	176.6 (38–733)	0–40
Bilirubin, mg/dL	6.4 (1.0–19.7)	0.0–1.2
Alkaline phosphatase, U/L	94 (58–172)	40–130
Creatinine, mg/dL	2.2 (0.8–4.7)	0.7–1.3

### Diagnosis and Treatment

All 8 patients had blood smears that were positive for identification of intraerythrocytic *Babesia* organisms (Table 4). Most (6/8) patients had an initial parasitemia of >10% (average parasitemia 18%). Three patients had PCR results obtained, all of which were positive. Six of the 8 patients had serologic test results obtained, and serum samples from all 6 patients were reactive for IgG (titers >1:256). 

**Table 4:** Diagnostic results for 8 patients with confirmed babesiosis, Penn State Health Milton S. Hershey Medical Center, Hershey, Pennsylvania, USA, 2005–2018

Patient no.	Smear (% parasitemia)	PCR result	IgG*	Co-infection with *Borrelia burgdorferi*
1	Positive (12)	Not obtained	Not obtained	Yes
2	Positive (45)	Positive	Positive	Yes
3	Positive (40)	Positive	Positive	Yes
4	Positive (11)	Not obtained	Positive	No
5	Positive (16)	Not obtained	Not obtained	No
6	Positive (17)	Positive	Positive	Yes
7	Positive (2)	Not obtained	Positive	No
8	Positive (1)	Not obtained	Positive	No

*IgG serologic test result considered positive if titer >1:256.

Concurrent Lyme disease was noted in half (4/8) of patients. Patients were screened for Lyme disease by using ELISA; if the result was positive, then a Western blot was performed. Patients had Lyme disease diagnosed if they had positive ELISA results and positive IgM or IgG results on Western blot. 

Most (7/8) patients received a combination of azithromycin and atovaquone for treatment. Three patients received clindamycin and quinine as part of their treatment; of these 3 patients, 1 patient received clindamycin and quinine alone for the duration of their therapy, and 2 patients were switched to azithromycin and atovaquone because of persistent parasitemia. Two of the patients who received clindamycin and quinine (1 of whom was switched to azithromycin and atovaquone) also required blood or platelet transfusions. Five patients underwent red cell exchange transfusions. The average duration of treatment was 18.1 days. The average duration of parasitemia was 9 days, but we only had exact date of clearance for 3 of the 8 patients.

### Hospital Course and Complications

Patients were identified as having severe babesiosis if they had parasitemia >10% or if they had intensive care unit (ICU) care, exchange transfusion requirement, intubation, acute respiratory distress syndrome, shock, or dialysis (*4*,*6*,*13*). Six of the 8 patients were classified as having severe infection. These 6 patients all had parasitemia >10%. Five of the 8 patients required ICU care and underwent exchange transfusions. One patient required dialysis, and 2 patients required blood or platelet transfusion. No patients required intubation or pressor support, and all patients survived. 

Four of the 6 patients with severe infection had co-infection with *Borrelia burgdorferi* (Lyme disease). The 2 nonsevere patients did not have co-infection. All 6 of the severely ill patients had infectious disease consults during their hospitalization. The 2 patients that were considered not severely ill did not receive an infectious disease consultation. The average length of stay for all patients was 11 days. The average length of stay for patients with severe infection was 12.3 days and for patients with nonsevere infection was 7 days.

## Discussion

**Figure 2:**
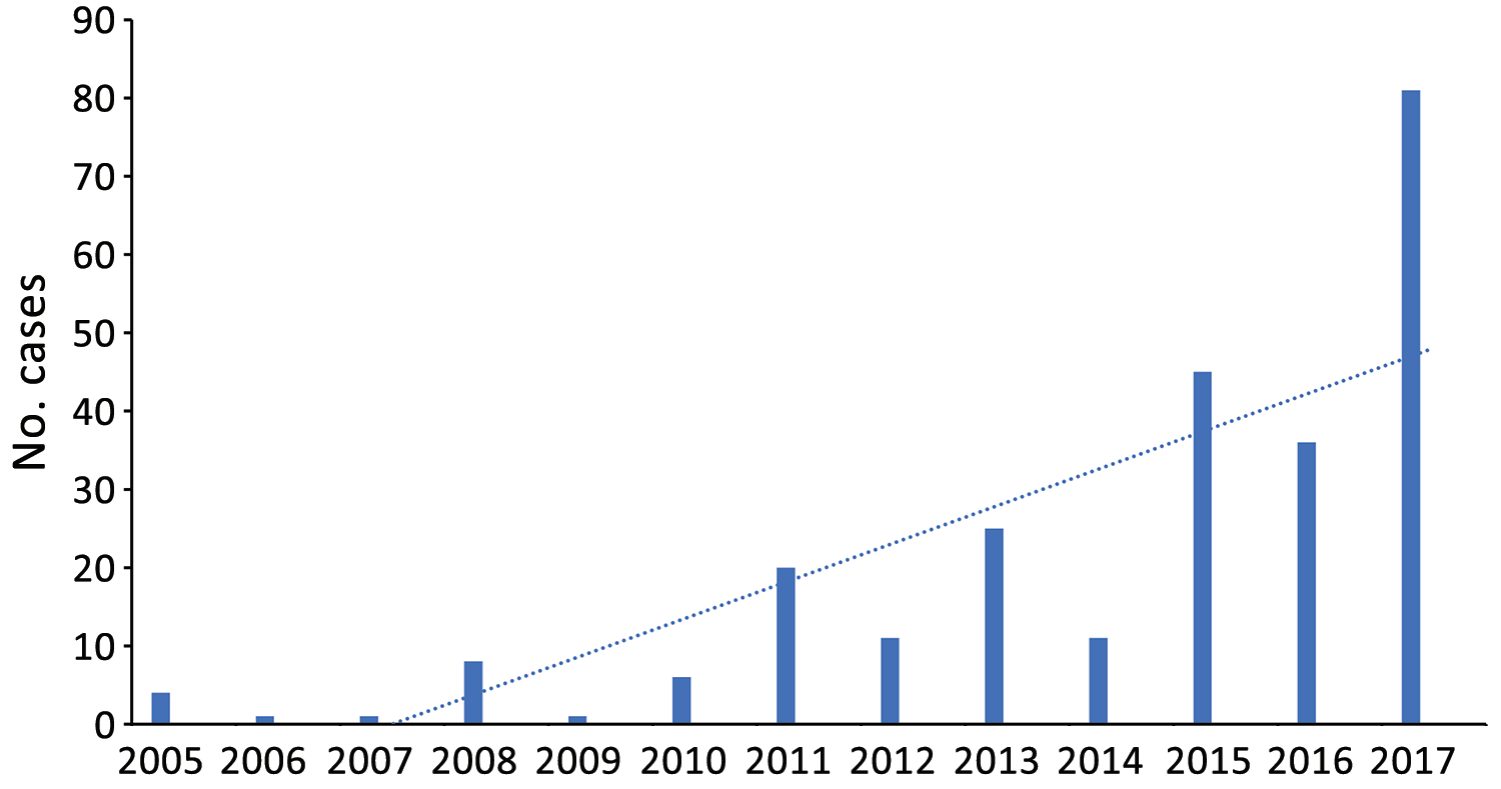
Annual number of babesiosis cases reported in Pennsylvania, USA, 2005–2017. Includes confirmed cases during 2005–2010 based on identification of *Babesia microti* organisms on blood smear and confirmed and probable cases reported during 2011–2017 based on the 2011 Centers for Disease Control and Prevention case definition (https://wwwn.cdc.gov/nndss/conditions/babesiosis/case-definition/2011). Dotted line indicates upward trend of cases over time.

Our findings further suggest that babesiosis is an emerging infection in the state of Pennsylvania. Data from our institution as well as the Pennsylvania Department of Health show a clear trend toward increasing cases throughout the state (Figure 2). In our study, 7 of the 8 reported cases were thought to be acquired in Pennsylvania. There were no reported cases during 2005–2010 and 1 case in 2011, followed by a steady rise in cases until 2018, when our institution saw 4 cases. The distribution of the areas of suspected infection acquisition makes us question whether we might be seeing a further expansion into central Pennsylvania over time. Over half (4/7) of the cases seen at Hershey Medical Center were acquired in southcentral Pennsylvania counties, and all 4 of those cases were seen most recently in the year 2018 (contrary to previous years, when cases occurred in northeast and southeast counties). These southcentral counties were determined by the home address ZIP codes of infected patients who had no history of travel before seeking care. Previous studies highlighting cases of babesiosis in Pennsylvania included patients from northeast and southeast counties only (*2*–*4*; Figure 1). Whether a westward expansion of babesiosis is truly occurring within the state is difficult to conclude on the basis of our small sample size; nonetheless, these findings warrant further inquiry, and we would be interested to see if other institutions in the south-central region have noted a similar trend. Because most persons infected with *B. microti* are either asymptomatic or have mild symptoms and diagnostic testing is often not obtained, the actual number of babesiosis cases at our institution was most likely underrepresented. In addition, given our methodology of focusing specifically on babesiosis-related codes in ICD-9 and ICD-10, we might have missed patients that were presumed to be co-infected (i.e., having both Lyme and babesiosis) and treated empirically.

Babesiosis is currently considered endemic in the US states of Connecticut, Massachusetts, Minnesota, New Jersey, New York, Rhode Island, and Wisconsin (*9*). Surveillance data shows a steady rise in babesiosis cases throughout the United States and further geographic expansion (*9*,*13*). When examining neighboring states that are currently endemic for babesiosis, specifically New York and New Jersey, we observed a historical pattern of expansion that might be indicative of what is to come in the state of Pennsylvania. Both states were endemic for Lyme disease, which shares the same vector as babesiosis (blacklegged ticks), before seeing the rise in babesiosis cases (*14*–*20*).

One study suggests that geographic spread of *B. microti* is favored by prior establishment of *B. burgdorferi* (the agent responsible for Lyme disease) and that co-infection (in mice reservoirs) with *B. burgdorferi* increases the likelihood of *B. microti* transmission (*21*). A paper from 1998 documented the presence of *I. scapularis* ticks in 49 of the 67 counties in Pennsylvania (*22*); however, a more recent study from 2015 identified the presence of the tick in all 67 counties (*10*). This same study reported tick infection rates of 47.4% for *B. burgdorferi*, 3.5% for *B. microti*, and 3.3% for *Anaplasma phagocytophilum*. This 3.5% infection rate by *Babesia* was higher than a previously reported rate of 0.7% in a 2010 report (*23*). The reasons for this suspected expansion are thought to be multifactorial, including the results of climatic effects on tick populations, growth of deer populations, and incursion into tick and deer habitats by humans (*13*).

When evaluating the patients treated at Hershey Medical Center, we found that our patients were demographically similar to patients described in prior studies; most patients in our investigation were male (75%) and elderly (median age 70 years). Only 1 patient reported a history of tick bite preceding infection, but most (75%) reported a history of outdoor activity. This finding further stresses the importance of having a high clinical suspicion in elderly patient populations despite absence of tick bite history.

The average number of days of symptoms before diagnosis was ≈10 days, which is a slightly shorter period compared with reports from other studies (*5*,*24*) and might have been because all patients had blood smears obtained early on during admission. The reason for obtaining blood smears was predominately for work-up of new anemia or thrombocytopenia. Most (75%) patients were identified as having severe infection; 6 of the 8 had parasitemia >10%. Five of these 6 patients required ICU care and underwent exchange transfusion. The purpose of ICU care in these patients was for close monitoring and exchange transfusion. No patients required pressor support or mechanical ventilation. The exchange transfusion process consists of removing a patient’s red blood cells and replacing them with donor red blood cells and is recommended for babesiosis patients who have parasitemia ≥10%; severe hemolysis (hemoglobin <10 g/dL); or pulmonary, hepatic, or renal impairment (*25*). No randomized trials have evaluated the efficacy of red cell exchange therapy; recommendations for this treatment are based on case series that show that parasitemia can be reduced by >50%–90% with red cell exchange therapy (*26*–*28*). Of the patients who required ICU care and exchange transfusion, only 1 of the patients was considered immunocompromised because of history of splenectomy; 1 other patient with severe infection had a history of splenectomy but did not require exchange transfusion. The remaining patients were considered immunocompetent, including 1 patient with diabetes. The unifying risk factor for most patients was older age. The high percentage of patients with severe infection was attributed to our hospital being a large tertiary academic center that received referrals from other hospitals. Some of the cases were referred specifically for evaluation for exchange transfusion. Co-infection with *B. burgdorferi* might have also contributed to severity of infection, given that 4 of the 6 patients with severe infection had serologic test results indicative of Lyme disease. Prior studies have shown increased disease severity and duration of illness in patients co-infected with *B. burgdorferi* and *Babesia* (*29*), which is consistent with the findings in our study. 

No deaths occurred in our study cohort. Average length of hospital stay was 11 days. Most patients (7/8) received treatment with azithromycin and atovaquone. Three patients received clindamycin and quinine; 1 patient received clindamycin and quinine alone for the duration of their therapy, and 2 patients were switched to azithromycin and atovaquone because of persistent parasitemia. These 2 patients improved with the azithromycin and atovaquone regimen; however, this outcome might have been attributable to their having undergone exchange transfusion. Historically, clindamycin and quinine was the regimen of choice for the treatment of *B. microti* infection (*1*). Later, azithromycin and atovaquone became recommended for mild to moderate disease because the regimen was shown to be as effective as the combination of clindamycin and quinine and had fewer adverse effects (*25*). Most recently, a study from 2017 suggested that azithromycin plus atovaquone was equally effective for patients with severe infection (*30*). 

## Conclusions

Ours is yet another article highlighting the emergence of babesiosis in the state of Pennsylvania. Given the nonspecific signs and symptoms associated with the illness and the potential severity of infection, especially in our elderly population, we believe that increased awareness and reporting of this infection is necessary. Clinicians must maintain a high index of suspicion in patients with a nonspecific febrile syndrome despite absence of tick bite history or lack of an immunocompromising condition. Evaluation for co-infections, particularly co-infection with *B. burgdorferi*, should be considered given patients with co-infection appear to have more severe disease.
